# Incorporating higher-order representative features improves prediction in network-based cancer prognosis analysis

**DOI:** 10.1186/1755-8794-4-5

**Published:** 2011-01-12

**Authors:** Shuangge Ma, Michael R Kosorok, Jian Huang, Ying Dai

**Affiliations:** 1 School of Public Health, Yale University, New Haven, CT, USA; 2 Department of Biostatistics, University of North Carolina at Chapel Hill, Chapel Hill, NC, USA; 3 Departments of Statistics and Actuarial Science, and Biostatistics, University of Iowa, Iowa City, IA, USA

## Abstract

**Background:**

In cancer prognosis studies with gene expression measurements, an important goal is to construct gene signatures with predictive power. In this study, we describe the coordination among genes using the weighted coexpression network, where nodes represent genes and nodes are connected if the corresponding genes have similar expression patterns across samples. There are subsets of nodes, called modules, that are tightly connected to each other. In several published studies, it has been suggested that the first principal components of individual modules, also referred to as "eigengenes", may sufficiently represent the corresponding modules.

**Results:**

In this article, we refer to principal components and their functions as representative features". We investigate higher-order representative features, which include the principal components other than the first ones and second order terms (quadratics and interactions). Two gradient thresholding methods are adopted for regularized estimation and feature selection. Analysis of six prognosis studies on lymphoma and breast cancer shows that incorporating higher-order representative features improves prediction performance over using eigengenes only. Simulation study further shows that prediction performance can be less satisfactory if the representative feature set is not properly chosen.

**Conclusions:**

This study introduces multiple ways of defining the representative features and effective thresholding regularized estimation approaches. It provides convincing evidence that the higher-order representative features may have important implications for the prediction of cancer prognosis.

## Background

In cancer research, high-throughput profiling has been extensively conducted, searching for genomic signatures with predictive power for traits or clinical outcomes. In this article, we analyze cancer prognosis studies, where the clinical outcomes are metastasis-free, overall, or other types of survival. We focus on microarray gene expression studies but note that the proposed approach is also applicable to data generated using other profiling techniques. In recent studies, gene signatures have been constructed for the prognosis of breast cancer, lymphoma, ovarian cancer, and cancers of many other organs [1].

The construction of molecular signatures for cancer prognosis has been investigated in many studies. This study complements and significantly advances from existing studies along the following directions. First, it accounts for the coordination among genes using the weighted coexpression network, whereas many existing studies ignore such coordination and assume the interchangeability of genes. Second, with properly constructed representative features, the proposed approach can accommodate the second-order effects of genes, whereas in many existing studies, only the linear effects of genes are considered. More importantly, this study provides convincing evidence that the higher-order representative features, which have often been neglected, can improve the predictive power. Thus, this study provides a way to improve over existing methodologies for the construction of prognosis signatures.

In the analysis of cancer genomic data, dimension reduction or feature selection is usually needed along with model estimation. Feature selection methods target at selecting a subset of genes, whereas dimension reduction methods construct a small number of representative features (sometimes referred to as "super genes" or "latent genes" in the literature) using the linear combinations of all genes. The approach developed in this article belongs to the family of dimension reduction approaches. Published studies have shown that the performance of feature selection and dimension reduction methods is data-dependent with no one dominating another [2,3].

Many existing analysis methods assume the interchangeability of gene effects and ignore the interplay among them. Extensive biomedical studies have shown that there is an inherent coordination among genes and, essentially, all biological functions of living cells are carried out through the coordinated effects of multiple genes [4,5]. Gene pathways and networks are perhaps the two most effective ways to describe the coordination among genes. Compared with pathway-based analysis, network-based analysis may have the following advantages. First, network-based analysis can use all the genes, whereas pathway-based analysis uses only annotated genes. Since many genes are not or only partially annotated, network-based analysis can be more comprehensive than pathway-based analysis. Second, in network-based analysis, the "distances" between genes are weighted (i.e., continuous measurements). Unlike in pathway-based analysis, we can infer not only whether two genes are connected but also the strength of connectedness. In this article, we focus on network-based analysis and defer a comprehensive comparison of pathway- and network-based methods to future studies.

In network analysis, nodes represent genes. Nodes are connected if the corresponding genes have similar biological functions and/or similar expression patterns across samples. There are subsets of nodes called "modules" that are tightly connected to each other. In this article, we adopt the weighted coexpression network (http://www.genetics.ucla.edu/labs/horvath/CoexpressionNetwork), which is built on the understanding that the coordinated coexpressions of genes encode interacting proteins with closely related biological functions and cellular processes [6]. Extensive empirical studies have shown that modules in the weighted coexpression networks often have important biological implications. Moreover, genes within the same modules tend to have related biological functions [7,8].

In cancer prognosis studies, the sample sizes are often small. With some modules having large sizes, dimension reduction is needed when conducting module-based network analysis. The approach developed in [9,10] and references therein proceeds as follows. First, principal component analysis is conducted within each module separately. Second, the first principal components, referred to as "eigengenes" in the literature, are identified. Following [11] and others, we refer to the principal components as "representative features". Third, the representative features are used as covariates in downstream model-building. There has been a rich literature on principal component analysis in gene expression studies [1,11-13]. Of note, many of those studies, for example [13], also recommend using only the first principal components.

In this article, for cancer prognosis studies with microarray gene expression measurements, we use the weighted coexpression networks and corresponding modules to describe the coordination among genes. Building on existing eigengene-based studies, we investigate the effects of higher-order representative features, which include principal components other than the first ones and second-order terms (quadratics and interactions). This study significantly differs from published ones. Specifically, unlike [1] and others, it is conducted in the context of network analysis, which may provide further insights into cancer biology beyond gene- and pathway-based analyses. Unlike in [9,10], higher-order representative features are investigated and shown to have important implications for prediction. In addition, since the dimensionality of representative features considered in this study is considerably higher than that in previous studies, regularized estimation is conducted. Unlike in [11], gene modules (as opposed to pathways) are the functional units. More importantly, this study considers the joint effects of multiple modules, whereas [11] studies the marginal effects.

## Methods

### Data and Model

#### Construction of weighted coexpression network and its modules

Besides the weighted coexpression network, there are other ways of constructing gene networks. Examples include the Boolean network, the Bayesian network, use of continuous models, and others. Compared with other networks, the weighted coexpression network has the following advantages. First, it uses only gene expression measurements and does not require any additional biological experiments. Second, it is computationally simple and can be constructed using existing software. And third, quite a few published studies have shown that it has satisfactory empirical performance [7,8]. On the other hand, it may also have limitations. The network is defined based on the correlations among gene expressions, which may not contain all information on the coordination among genes. In addition, the network construction is unsupervised. In this article, we focus on the weighted coexpression network and defer the comparison of different networks to future studies.

Construction of the weighted coexpression network consists of the following steps.

1. For genes *k *and *j *(= 1... *d*), compute *cor *(*k, j*), the Pearson correlation coefficient of their expressions. Compute the similarity measure *S*(*k, j*) = |*cor*(*k, j*)|;

2. Compute the adjacency function *a_k,j _*= *S^b^*(*k, j*), where the adjacency parameter *b *is chosen using the scale-free topology criterion;

3. For gene *k *(= 1 ... *d*), compute its connectivity $C_{k}=\sum_{u=1}^{d}a_{k,u}$;

4. For genes *k *and *j *(= 1 ... *d*), compute the topological overlap based dissimilarity measure *d_k,j _*= 1 *- ω_k,j _*where *ω_k,j _*= (*l_k,j _*+ *a_k,j_*)/(*min*(*C_k, _C_j_*) + 1 *- a_k,j_*) and $l_{k,j}=\sum_{u=1}^{d}a_{k,u}a_{j,u}$. Define the dissimilarity matrix *D*, whose (*k, j*)th element is *d_k,j_*;

5. Conduct hierarchical clustering with matrix *D*. Apply the dynamic tree cut approach to cut the clustering tree (dendrogram) and identify the resulting branches as gene modules [14].

In Steps 1 and 2, the adjacency measure between genes is defined using the power transformation of correlation coefficients. We adopt the weighted network, which can measure not only whether two genes are connected but also the strength of connection. The power adjacency function has the attractive factorization property. In our study, we find that for the six datasets analyzed, *b *= 6 (which has been suggested in published studies) can lead to results nearly satisfying the scale-free topology criterion. This criterion has been motivated by the observation that metabolic networks exhibit scale-free topology. In Step 4, we use the topological overlap dissimilarity measure [15], which has been found to result in biologically meaningful modules. In addition, this measure is relatively robust in describing the interconnectedness between genes [8]. The advantage of the dynamic tree cut approach in Step 5 has been discussed in [14].

#### Construction of representative features

Assume that *M *modules have been constructed. For module *m *(= 1 ... *M*), denote *m_i _*as the number of genes within this module and $U_{1}^{m}...\;U_{m_{i}}^{m}$ as the gene expressions. With principal component analysis, any linear combination of *U*s can be written as

$$\alpha_{1}U_{1}^{m}+...+\alpha_{m_{i}}U_{m_{i}}^{m}=\gamma_{1}X_{1}^{m}+...+\gamma_{m_{k}}X_{m_{k}}^{m},$$

where $X_{1}^{m}...\;X_{m_{k}}^{m}$ are the *m_k _*PCs and *m_k _*is the rank of $\left(U_{1}^{m},....,U_{m_{i}}^{m}\right)$. In particular, $X_{i}^{m}\text{s}$ have unit norms and are the linear combinations of $U_{i}^{m}\text{s}$, $X_{i}^{m}$ and $X_{j}^{m}$ are orthogonal to each other when *i *≠ *j*. Variation explained by $X_{i}^{m}$ decreases as *i *increases.

Several studies propose using $\left(X_{1}^{1},...,X_{1}^{M}\right)$ (i.e., the first principal components from the *M *modules) as covariates in downstream analysis [9,10,13]. In this study, we are interested in not only the first principal components but also the other principal components, as well as quadratics and interactions of principal components. Specifically, we consider the following four sets of representative features:

(R1) Consider $\left\{X_{1}^{1},...,X_{1}^{M}\right\}$. That is, the first principal components from all modules. This set of *M *representative features has been investigated in previous studies and will serve as a benchmark. To unify notations, denote *Z*_0, *i *_= *X*_1_*^i ^*(*i *= 1 ... *M*) and *Z *= (*Z*_0,1_*, ..., Z*_0,*M*_);

(R2) Denote *Z*_0,*i *_= *X*_1_*^i ^*(i = 1... M). For 1≤ *i≤ j≤ M*, define *Z_i,j _*= *Z*_0,*i *_× *Z*_0,*j*_. Consider *Z *= (..., *Z*_0,*i*_*,.., Z_i,j_*,...). This set of representative features is composed of the first principal components from all modules, their quadratics, and their second order interactions;

(R3) Within module *m *(= 1,...,*M*), select the top m* principal components such that *ξ*% of the total variation of gene expressions is explained [16]. Define $P=\sum_{m=1}^{M}m*$. Consider $Z=(Z_{0,1},...,Z_{0,P})=(X_{1}^{1},...,X_{1}^{1},...X_{1}^{M},...,X_{M}^{M}{}_{*})$. This set of representative features is composed of principal components that can sufficiently explain the variation of gene expressions. In our study, we set *ξ*% = 80%, which is slightly smaller than that adopted in [16]. Our data analysis suggests that, because of the extremely noisy nature of gene expressions, a huge number of principal components are needed to explain, for example, 95% of the variation;

(R4) We first construct the *P *principal components as with (R3). Denote $\left(Z_{0,1},...,Z_{0,P})=(X_{1}^{1},...,X_{1}^{1},...X_{1}^{M},...,X_{M}^{M}{}_{*}\right)$. For 1≤ *i *≤ *j≤ P *, define *Z_i,j _*= *Z*_0,*i *_× *Z*_0,*j*_. Consider *Z *= (..., *Z*_0,*i*_*,..., Z_i,j_,..*.), the set composed of the *P *principal components and their quadratics.

Among the above sets of representative features, (R1) has been investigated elsewhere and will be used as a benchmark. With (R3), we analyze multiple principal components per module. In the detection of marginally differentially expressed pathways, Ma and Kosorok [11] show that higher-order principal components may have important implications. With (R2) and (R4), we are able to account for the interactions among genes within the same modules. More importantly, we are able to account for the interactions among different modules using their principal components. The relatively small number of principal components per module makes it possible to study the interactions, which are extremely difficult to study in gene-based analysis.

Following a similar spirit, there may be other ways of defining the representative features. For example, for module *m*, it is possible to accommodate the interactions among genes by conducting principal component analysis with the set $\left\{U_{i}^{m}:1\leq i\leq m_{i}\}\cup \{U_{i}^{m}\times U_{j}^{m}:1\leq j\leq m_{i}\right\}$. However, when the module sizes are large, such construction may be computationally expensive. Our exploration suggests that (R1)-(R4) are the relatively simpler and more intuitive ways of constructing the representative features.

#### Statistical modeling

Denote *T *and *C *as the survival and random censoring times, respectively. We assume that the gene expressions are associated with cancer survival through the Cox proportional hazards model, where the conditional hazard function is λ(*t|Z*) = λ_0_(*t*) exp(*β' Z*). Here λ_0_(*t*) is the unknown baseline hazard, and *β *is the unknown regression coefficient. Under right censoring, one observation consists of (*Y *= *min*(*T, C*), Δ = *I*(*T≤C*), *Z*). Assume *n *iid observations (*Y_i_, δ_i_*, *Z_i_*)*, i *= 1 ... *n*. Denote *r_i _*= {*k*: *Y_k _≥ Y_i_*} as the at-risk set at time *Y_i_*. The log-partial likelihood function is $R(\beta )={\sum_{i}\delta }_{i}\{\beta^{\prime }Z_{i}-\log(\sum_{k\in r_{i}}\exp(\beta^{\prime }Z_{k}))\}$. Here we describe the relationship between genes and cancer survival using the representative features. As the representative features are functions of genes and their second-order terms, we can rewrite the Cox models in terms of genes.

### Regularized estimation

Although the dimensionality of *Z *is expected to be smaller than that of the original gene expressions, it may still be comparable to or even larger than the sample size, particularly with (R2)-(R4). In addition, it is possible that only a subset of the representative features is associated with cancer survival. Thus, we consider regularized estimation, which can effectively "stabilize" estimation and discriminate cancer-associated representative features from noises. With (R1) and (R3), we use the TGDR (Threshold Gradient Directed Regularization) approach [17]. As shown in [17] and follow-up studies, TGDR has a lower computational cost and empirical performance comparable to or better than that of alternative methods. With (R2) and (R4), we modify the TGDR to better accommodate the second-order terms. Particularly, under the modified approach, when a second-order term is included in the model, its corresponding first-order terms are automatically included.

#### The TGDR algorithm

The TGDR approach can be used for regularized estimation when representative features (R1) and (R3) are adopted. Let Δ*ν *be a small positive increment. In numerical studies, we set Δ*ν *= 10^-3^. Denote 0 ≤ *τ *≤ 1 as the threshold. The TGDR algorithm consists of the following steps.

1. Initialize *β *= 0;

2. With the current estimate of *β*, compute the vector of gradient *g *= ∂*R*(*β*)/∂*β*. Denote the *j*th element of *g *as *g_j_*;

3. Compute the thresholding vector *f*, where its *j*th element is *f_j _*= *I*(|*g_j_| ≥ τ *× *max_l_|g_l_|*);

4. Update the estimate *β_j _*= *β_j _*+ Δ*ν *× *g_j _*× *f_j_*;

5. Iterate Steps 2-4 *K *times, where *K *is determined via cross validation.

#### The modified TGDR algorithm

When representative features (R2) are (R4) are used, the following algorithm can better accommodate the

second order terms.

1. Initialize *β *= 0;

2. With the current estimate of *β*, compute the vector of gradient *g *= ∂*R*(*β*)/∂*β*. Denote *g_i,j _*as the component of *g *that corresponds to *Z_i,j_*;

3. Compute the thresholding vector *f*. Denote *f_i,j _*as its component corresponding to *Z_i.j_*. Define

(1) $$f_{i,j}=\{\begin{array}{ll} I(|g_{i,j}|\geq \tau \times max_{l,u}|g_{l,u}|) & \text{for}\;i>0,j>0\\ I(|g_{i,j}|\geq \tau \times max_{l,u}|g_{l,u}|) & \\ \text{OR}\sum_{u}f_{i,u}>0\;\text{OR}\sum_{u}f_{j,u}>0) & \text{for}\;i=0,j=0. \end{array}$$

4. Denote *β_i,j _*as the component of *β *that corresponds to *Z_i,j_*. Update the estimate *β_i,j _= β_i,j_+*Δν *× g_i,j _× f_i,j_*,

5. Iterate Steps 2-4 *K *times, where *K *is determined via cross validation.

#### Remarks

The above two approaches use thresholding for regularized estimation and feature selection. Specifically, at each iteration, the gradients, which measure the relative importance of representative features, are computed. More important representative features tend to have larger gradients. Only the important representative features are selected, and their estimated regression coefficients are updated. The iteration stops when a cross validation-based criterion is reached. The second algorithm ensures that, if a second-order term is selected, its corresponding first-order terms are selected. This cannot be automatically achieved with the first algorithm. We refer to [17] for a more detailed discussion of thresholding regularization. Both approaches involve tuning parameters *τ *and *K*, which are selected using V-fold cross validation. In data analysis, we set *V *= 5 and search over *τ *= 1.0, 0.95, 0.9, ..., 0.05, 0. Research code written in R is available at http://www.med.yale.edu/eph/faculty/labs/ma/ for the construction of network modules and representative features and regularized estimation.

## Results

### Analysis of cancer prognosis studies

#### Data collection

We collect six cancer prognosis studies with microarray measurements. We refer to them as data D1-D6 and provide brief descriptions below and in Table 1.

**Table 1:** Description of datasets.

Data	Disease	Platform	Gene	Sample
D1: Rosenwald et al. (2003)	MCL	cDNA	8810	92
D2: Dave et al. (2004)	FL	Affymetrix	44928	187
D3: Rosenwald et al. (2002)	DLBCL	cDNA	7399	240
D4: Sotiriou et al. (2003)	Breast cancer	cDNA	7650	98
D5: van't Veer et al. (2002)	Breast cancer	Oligonucleotide	24481	78
D6: Huang et al. (2003)	Breast cancer	Affymetrix	12625	71

Gene/Sample: number of genes/subjects profiled.

**D1**. A study using microarray expression analysis of mantle cell lymphoma (MCL) was reported in [18]. Among 101 untreated patients with no history of previous lymphoma, 92 were classified as having MCL based on established morphologic and immunophenotypic criteria. Survival times of 64 patients were available, and the other 28 patients were censored. The median survival time was 2.8 years (range 0.02 to 14.05 years). Lymphochip DNA microarrays were used to quantify mRNA expressions in the lymphoma samples from the 92 patients. Gene expression data on 8,810 cDNA elements was available.

**D2**. A study was conducted to determine whether the survival risk of patients with follicular lymphoma (FL) can be predicted by gene expression profiles of the tumors [19]. Fresh-frozen tumor biopsy specimens from 191 untreated patients who had received a diagnosis of follicular lymphoma between 1974 and 2001 were obtained. The median age at diagnosis was 51 years (range: 23 to 81), and the median follow-up time was 6.6 years (range: less than 1.0 to 28.2). The median follow-up time among patients alive at the last follow-up was 8.1 years. Eight records with missing survival information are excluded from analysis. Affymetrix U133A and U133B microarray genechips were used to measure gene expressions of 44,928 probes.

**D3**. Rosenwald et al. [20] reported a diffuse large B-cell lymphoma (DLBCL) prognosis study. This study retrospectively collected tumor biopsy specimens and clinical data for 240 patients with untreated DLBCL. The median follow-up was 2.8 years, with 138 observed deaths. A lymphochip cDNA microarray was used to measure the expressions of 7,399 genes.

**D4**. Sotiriou et al. [21] reported a study correlating gene expression measurements generated using cDNA with clinico-pathological characteristics and clinical outcomes in an unselected group of 99 node-negative and node-positive breast cancer patients. In the original analysis, the Cox model was used to identify genes that were marginally significantly associated with relapse-free survival. In this study, we analyze the 98 patients with complete survival information.

**D5**. Van't Veer et al. [22] reported a breast cancer prognosis study investigating the time to distant metastasis. Ninety-seven (97) lymph node-negative breast cancer patients 55 years old or younger participated in this study. Among them, 46 developed distant metastases within 5 years. Complete information was available for 78 subjects. Expression levels of 24,481 probes were measured.

**D6**. Despite major progress in breast cancer treatment, the ability to predict metastasis of the tumor remains limited. Huang et al. [23] reported a study investigating metastastic states and relapses in breast cancer patients. Affymetrix genechips were used for the profiling of 71 samples. Expression measurements on 12,625 probes were available.

Among the above studies, three used cDNA, one used oligonucleotide arrays, and two used Affymetrix genechips for profiling. We process each dataset separately as follows. We conduct microarray normalization using a lowess normalization approach for cDNA data and a robust normalization approach for Affymetrix data [24]. We impute missing measurements using the K-nearest neighbors approach. We select 2,000 genes with the largest variances for downstream analysis. Since we expect the number of genes associated with cancer prognosis to be far less than 2,000, and since we are more interested in genes with a high level of variation, we conduct this unsupervised screening to reduce computational cost. In addition, recent studies have shown that prescreening may improve feature selection accuracy [25]. We then rescale gene expressions to have zero median and unit variance.

#### Evaluation of prediction performance

Main objectives of cancer genomic studies include marker identification and predictive model-building. Despite the fast accumulation of knowledge on the biological functions of genes, there is still a lack of commonly accepted, objective ways of determining the accuracy and implications of identified markers. Thus, as in many published studies, we focus on the prediction performance of the models and identified representative features. It is expected that the evaluation of prediction performance can also provide an indirect evaluation of the biological implications of the models and representative features. For prognosis of some cancers, for example breast cancer, there are multiple independent studies. Thus, there is a possibility of making cross-study prediction. However, cross-study prediction demands comparability between studies [26]. Without having access to all the details on experimental set-up, we are unable to determine whether there are prognosis studies fully comparable to the six studies we analyze. Thus, in the study, we choose not to conduct cross-study prediction evaluation.

As an alternative, we consider a cross validation-based approach. We acknowledge that cross validation-based evaluation has a small chance of generating overly optimistic results. However, the adopted V-fold cross validation-based approach is expected to be reasonably objective. In addition, different sets of representative features are evaluated using the same approach. Thus, the evaluation results are expected to be meaningful. The cross validation-based evaluation proceeds as follows. (a) Randomly partition data into V subsets with equal sizes. In our numerical study, set *V *= 5; (b) For *υ *= 1 ... *V*, remove subset υ from data; (c) With reduced data, carry out the cross validation and regularized estimation. Denote the estimated regression coefficient as ${\hat{\beta }}^{\left(-\upsilon \right)}$ (d) Compute the predictive risk scores ${\hat{\beta }}^{\left(-\upsilon \right)}{}^{\prime }$*Z *for the removed subjects; (e) Repeat Steps (b)-(d) over all subsets; (f) Compute two summary statistics. (f.1) The first is the logrank statistic [27]. Dichotomize the predictive scores at the median. Create two risk groups. Compute the logrank statistic, which measures the difference of survival between the two groups. Under the Null, the representative features have no predictive power, and the logrank statistic is χ^2 ^distributed with degree of freedom 1. We compute the logrank statistic using the R function *survdiff*. (f.2) The second is the concordance index, which is computed using the R function *rcorr.cens*. A larger concordance index indicates better predictive power, with concordance index equal to 0.5 corresponding to random guess. This evaluation approach has been extensively used in cancer genomic studies.

#### Analysis results

For each dataset, we construct the weighted coexpression network and its modules. For dataset D1-D6, 12, 10, 13, 10, 11, and 13 modules are constructed, respectively. More details are available in Additional File 1. We then construct the four sets of representative features.

We conduct the cross validation-based prediction evaluation and present the logrank statistics and concordance indices in Table 2. We can see that, when (R1), the first principal components from all modules, are used, five out of the six logrank statistics are significant at the 0.05 level. This observation is in line with the satisfactory results observed in [9,10] and others. We also find that, the (R2)-(R4) logrank statistics can be larger than those of (R1), which suggests that prediction performance can be improved by incorporating higher-order representative features. The improvement is considerably large for dataset D2 and D4. Another finding is that the prediction performance of representative features (R2)-(R4) is data-dependent. Particularly, two datasets have (R2), two have (R3), and the other two have (R4) logrank statistics as the largest. Examining the concordance indices suggests reasonable predictive power of the representative features and similar conclusions as with the logrank statistics.

**Table 2:** Data analysis results: prediction logrank statistics and concordance indices.

	Logrank statistic				Concordance index			
Data	R1	R2	R3	R4	R1	R2	R3	R4
D1	15.30	19.10	**34.70**	0.18	0.74	0.70	**0.77**	0.50
D2	0.25	**4.56**	0.60	0.46	0.61	**0.67**	0.51	0.58
D3	10.40	0.32	**19.00**	2.14	0.62	0.53	**0.69**	0.55
D4	3.89	**13.80**	11.40	0.01	0.63	**0.66**	0.64	0.54
D5	7.95	7.50	7.50	**12.30**	0.72	0.70	0.70	**0.76**
D6	6.27	2.15	6.46	**7.99**	0.65	0.61	0.69	**0.73**

Larger logrank statistics and concordance indices correspond to more predictive power. A logrank statistic greater than 3.84 is significant at the 0.05 level.

To gain further insights, we also conduct the following analysis. The TGDR and modified TGDR algorithms are capable of selecting a small number of important representative features. For each dataset, we examine the selection results for the representative feature set with the largest logrank statistic. Detailed results are presented in Additional File 2. In addition, although the models are constructed using representative features, we can rewrite using genes and their second-order terms. Principal components are the linear combinations of all genes within specific modules. The statistical models we construct are sparse at the representative feature level but not at the gene level. We examine the top 20 genes and/or interactions of genes with the largest regression coefficients. Since all gene expressions have been normalized to have equal variances, the magnitude of regression coefficients can provide a rough measure of the relative importance of genes.

**D1**. The representative features (R3) are adopted. Six principal components (#2, 5, 6, 7, 9, 11) from module #2 and 1 principal component from module #9 are identified. It is interesting to note that in module #2, the first principal component is not identified. Among the 20 genes with the largest regression coefficients, there are established cancer markers, including for example genes PTK2, PCNA, and PRKACA. There are also new discoveries that need further investigation.

**D2**. The representative features (R2) are adopted. We identify 6 principal components, quadratics of 4 principal components, and 4 interactions among 5 principal components. We conclude that among the 10 modules, only 6 are cancer-associated. In addition, the interactions among modules have non-ignorable effects. We also examine the top 20 regression coefficients and find that 9 of them come from individual genes and 11 come from interactions of genes.

**D3**. The representative features (R3) are adopted. Five out of 13 modules are identified as associated with prognosis. More specifically, 15, 1, 4, 1, and 1 principal components are identified in module #2, 4, 5, 12, and 13, respectively. For all of the 5 identified modules, the first principal components are identified. We examine the 20 genes with the largest regression coefficients and find that quite a few belong to the MHC (major histocompatibility complex) family. Of note, we conduct probe-level (as opposed to gene-level) analysis, with the consideration that different probes may correspond to different segments of the same genes.

**D4**. The representative features (R2) are adopted. Eight out of 10 modules are identified as associated with prognosis. More specifically, we identify 8 principal components, quadratics of 3 principal components, and 10 interactions among 8 principal components. As with data D2, we also observe the nonzero effects of interactions among modules. When examining the top 20 regression coefficients, we find that 12 come from individual genes, 1 comes from the quadratic of a gene, and the remaining 7 come from interactions of genes.

**D5**. The representative features (R4) are adopted. Six out of 11 modules are identified as associated with prognosis. Among them, the first principal components are identified in 5 modules (all except module #5). We identify 18 principal components, quadratics of 8 principal components, and 12 interactions among 14 principal components. When examining the top 20 regression coefficients, we find that all of them come from individual genes. Among the top 20 genes, there are several known breast cancer markers, including for example genes IL8, N-myc, PRKA6, and others.

**D6**. The representative features (R4) are adopted. Three out of 13 modules are identified as associated with prognosis. The first principal component is identified in only 1 of the 3 modules. We identify 10 principal components, quadratics of 2 principal components, and 7 interactions among 9 principal components. When examining the top 20 regression coefficients, we find that all of them come from individual genes.

### Simulation

To better understand properties of the proposed representative features and regularized estimation approaches, we conduct simulation studies. As observed gene expressions usually do not fit specific parametric distributions [28], we randomly sample gene expressions of 200 subjects without replacement from D1-D3 combined (the lymphoma datasets). We use subjects as the sampling units so that the correlation structure among genes is kept. We then randomly split the 200 samples into a training set and a testing set, each with 100 subjects. We construct the weighted coexpression network, its modules, and 4 sets of representative features using the training set. With (R1)-(R4), respectively, we randomly select 10 representative features as associated with prognosis and set the rest as noises. The prognosis-associated representative features have their non-zero regression coefficients generated randomly from *Unif *[-0.5, 0.5]. The survival times are then generated from the Cox model with λ_0_(*t*) = 0.5 (i.e., constant baseline hazard). The censoring times are generated independent of survival. We adjust the censoring distribution so that the censoring rate is about ~ 40%. Thus, there are a total of 4 different data-generating models, with (R1)-(R4) being the "true" representative features.

With the training set, we use the representative features (R1)-(R4) and proposed regularized approaches for estimation. This step reflects the fact that, in practice, it is unknown which set of representative features is appropriate. We make predictions for subjects in the testing set using the training set estimates. The logrank statistic and concordance index are computed for evaluation of prediction performance. We note that, unlike in practical data analysis, in the above simulation, the training and testing sets are completely independent.

Summary statistics based on 500 replicates are shown in Table 3. Simulation suggests the importance of properly specifying the representative features. With data S1, where (R1) is the model generating representative features, all four sets of representative features can lead to satisfactory prediction performance. This observation is reasonable considering that (R1) is a subset of (R2)-(R4). Similar observations and reasonings hold for data S2 and S3. However, when (R4) is the true data generating representative features, results under (R1)-(R3) are significantly less satisfactory.

**Table 3:** Simulation study: mean prediction logrank statistics and concordance indices based on 500 replicates.

	Logrank statistic				Concordance index			
Data	R1	R2	R3	R4	R1	R2	R3	R4
S1	94.15	94.92	90.72	88.57	0.95	0.95	0.95	0.94
S2	4.92	59.62	7.32	82.03	0.60	0.88	0.62	0.93
S3	39.45	45.70	76.19	68.53	0.80	0.82	0.90	0.88
S4	2.27	29.09	4.54	80.57	0.57	0.79	0.60	0.93

Simulation seems to suggest that (R4), the most complicated set of representative features, is the proper choice under all four simulation scenarios. A drawback of (R4) is its high computational cost, particularly when there are a moderate to large number of modules, which may make it less appealing in practical data analysis. In addition, the simulation settings may still be overly simplified compared with what is observed. With real data, as can be seen from Table 2 (R4) is not necessarily dominatingly better. There are multiple reasons for the different patterns observed in Table 2 and 3. The first is that, with practical data, (R1)-(R4) do not necessarily include the true data-generating mechanisms. The second is that, with real data, there may not be a clear cut between signals and noises. Instead of a small number of large signals, there may be a large number of small signals. In addition, with simulated data, the survival is determined by gene signatures. In contrast, in practice, the survival may also be affected by other risk factors such as cancer treatment history, which explains the smaller predictive power observed in Table 2.

## Discussion

For cancer prognosis studies with gene expression measurements, we describe the interplay among genes using the weighted coexpression network and use principal component analysis techniques to reduce the dimensionality of gene expressions. This study complements and advances from existing studies by investigating the contribution of higher-order representative features to predictive power. The four sets of representative features investigated in this study share some desired properties with other principal components-based analysis. For example, the computational cost is affordable, and the majority of the variation of gene expressions can be accounted for.

As the dimensionality of representative features may be moderate to large, the TGDR and a modification of the TGDR are used for regularized estimation and feature selection. In [17] and several follow-up studies, it is shown that the TGDR has performance comparable to or better than that of existing alternatives. As the TGDR cannot automatically accommodate the second-order terms, a modification of the TGDR is necessary. Examination of Table 2 shows that some of the prediction logrank statistics and concordance indices are small, suggesting possible local optimums. We note that there are many available approaches that can be used for regularized estimation. For example, penalization approaches have attracted extensive attention in recent statistical and bioinformatic literature [2]. However, we note that most existing (including penalization) approaches may have a problem with local optimums. A satisfactory solution to this problem is highly challenging and warrants separate investigation.

In this study, the proposed research question is investigated using both real and simulated data. In data analysis, without data from independent comparable studies, we conduct cross validation-based prediction evaluation. Such an evaluation is expected to be reasonably fair. However, independent confirmation studies will be needed to fully validate the findings. With the 6 real datasets analyzed, 3 different sets of representative features have the best prediction performances. This finding is in line with [11] and is not surprising considering the extreme complexity and heterogeneity of cancer. Examination of individual regression coefficients suggests that different datasets may have significantly different scenarios. Particularly, for some datasets, the quadratics and interactions among genes may have important implications. Our investigation does not yield a way to suggest the "optimal" representative features. Our recommendation is that, in practical analysis, *researchers need to experiment with different sets of representative features*.

In some previous network-based analysis, geneset enrichment analysis has been conducted to investigate whether modules identified as associated with prognosis are enriched with certain pathways or represent certain biological processes. We note that such analysis is also possible in this study. However, consider a hypothetical module with only 2 principal components. Consider the following two different scenarios. Under scenario 1, the second principal component is identified as associated with prognosis. Under scenario 2, the first principal component and its quadratic are identified as associated with prognosis. Under both scenarios, this module is identified as associated with prognosis. However, an important goal of this study is to discriminate between those two scenarios. Considering that the enrichment analysis will lead to the same results under those two scenarios and thus can be misleading, we choose not to conduct enrichment analysis.

We have investigated second-order representative features. In a similar manner, it is possible to consider third- or even higher-order terms. Such an effort may considerably increase the dimensionality and computational cost. We construct the representative features in an unsupervised manner, which has low computational cost and can be easily implemented using existing software. In recent principal component analysis studies, it has been suggested that supervised methods may outperform unsupervised methods [13,29]. It is possible to construct the supervised counterparts of the proposed representative features.

## Conclusions

In this study, we propose using principal component analysis-based representative features for dimension reduction in weighted coexpression network analysis. The proposed representative features and TGDR regularized estimation provide an effective way of reducing the dimensionality, accounting for the interactions among genes within the same modules, and, more importantly, accounting for the interactions among modules. The investigation on the interactions may provide a useful addition to the literature. Our most important finding is that incorporating higher-order representative features leads to improved prediction performance, which may help build better predictive models for cancer prognosis.

## Competing interests

The authors declare that they have no competing interests.

## Authors' contributions

All authors were involved in the study design and writing. SM and YD conducted numerical studies. All authors read and approved the final manuscript.

## Pre-publication history

The pre-publication history for this paper can be accessed here:

http://www.biomedcentral.com/1755-8794/4/5/prepub

## Supplementary Material

Additional file 1 **Results on network module construction**. This additional file contains the details on the network modules constructed using WGCNA. Click here for file

Additional file 2 **Analysis results**. This additional file contains the detailed analysis results for dataset D1-D6. Click here for file
