# Supplementary material for: Incorporating higher-order representative features improves prediction in network-based cancer prognosis analysis
Source: BMC Med Genomics. 2011 Jan 12;4:5. doi: 10.1186/1755-8794-4-5 (PMC3037289; doi:10.1186/1755-8794-4-5)

Dataset D1: Rosenwald et al. (2003)


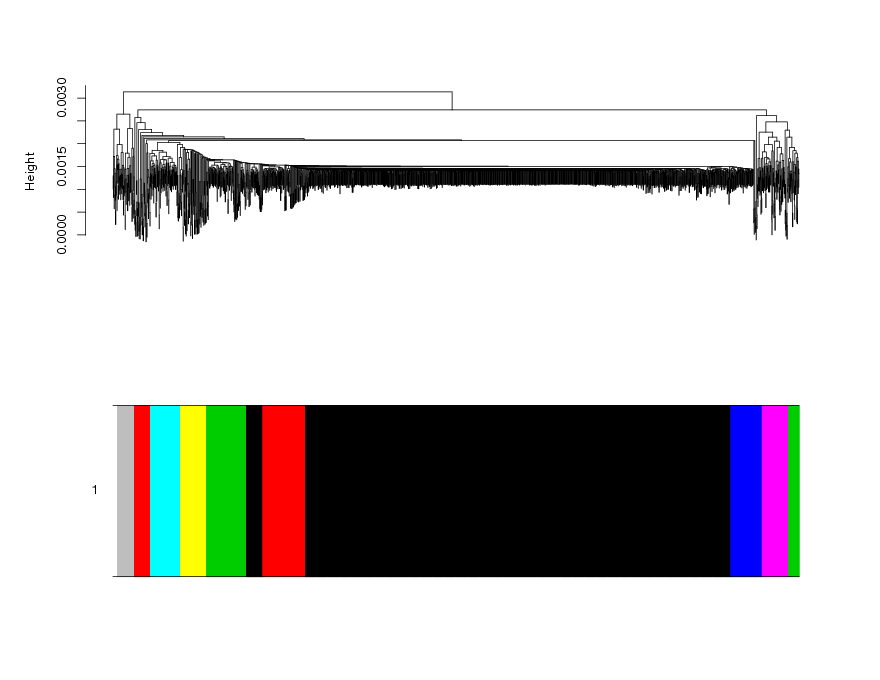


Dataset D2: Dave et al. (2004)


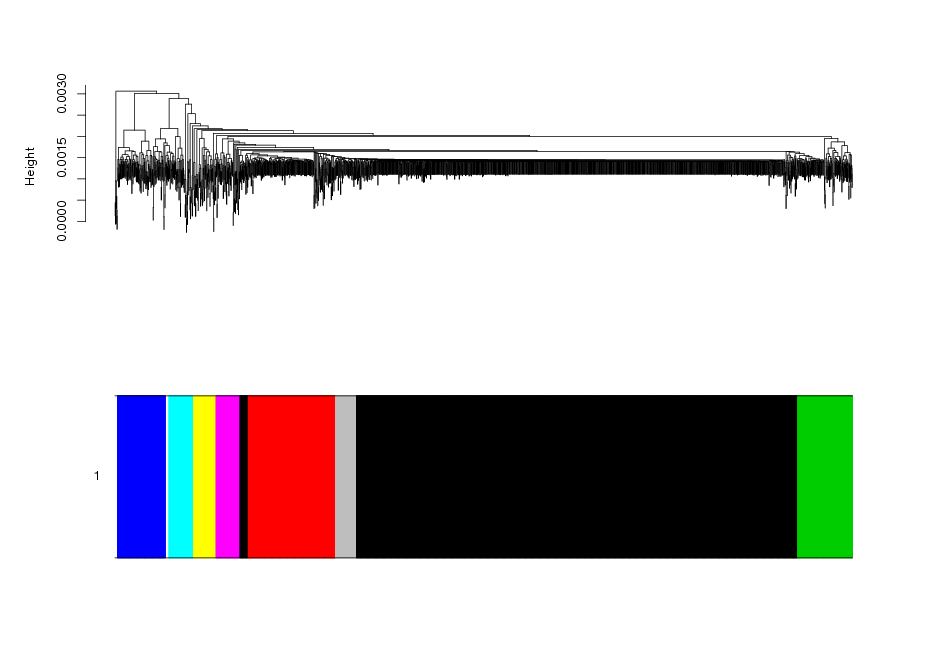


Dataset D3: Rosenwald et al. (2002)


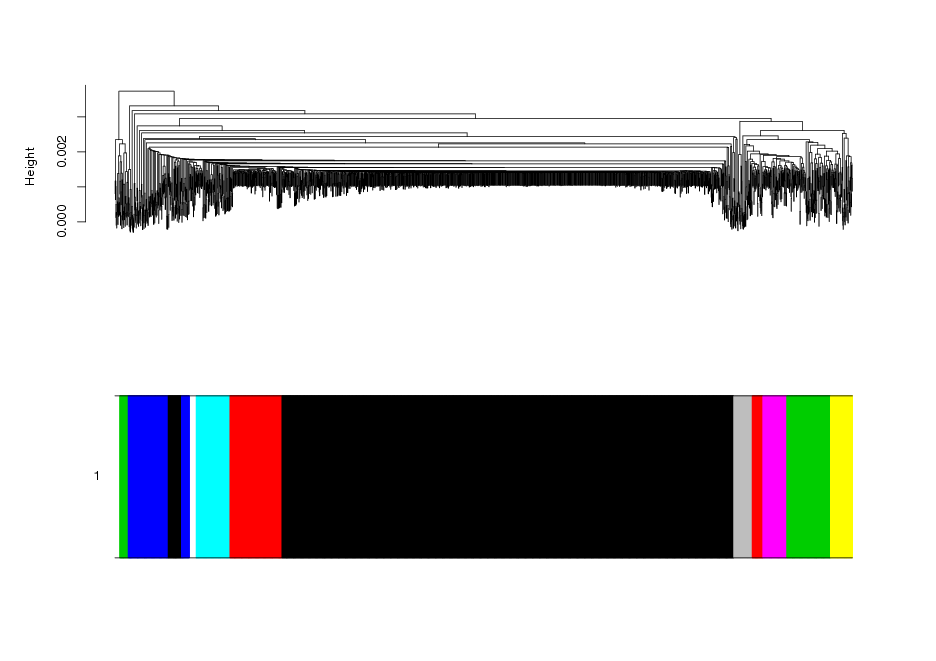


Dataset D4: Sotiriou et al. (2003)


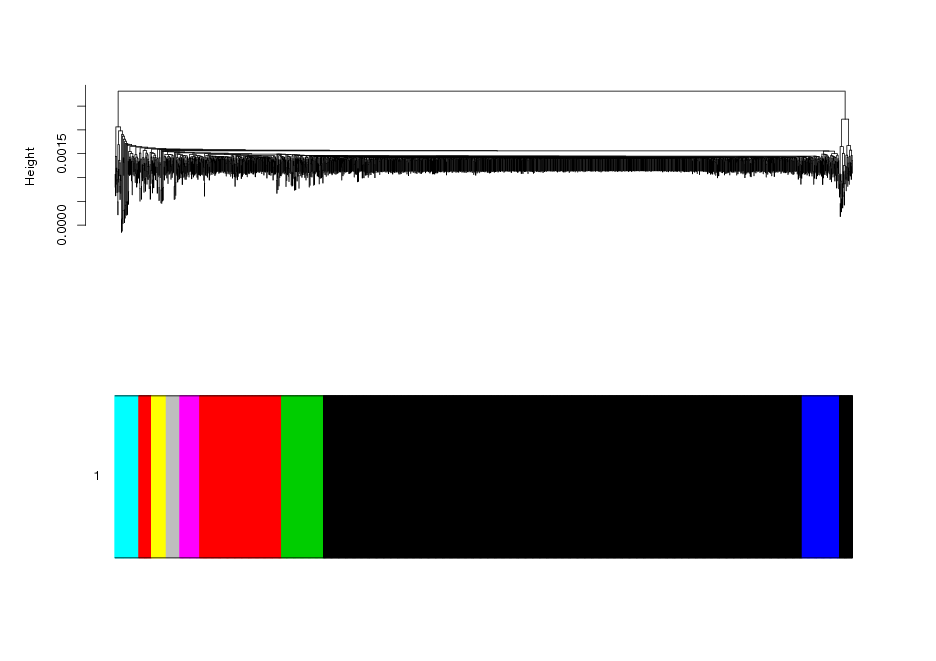


Dataset D5: van’t Veer et al. (2002)


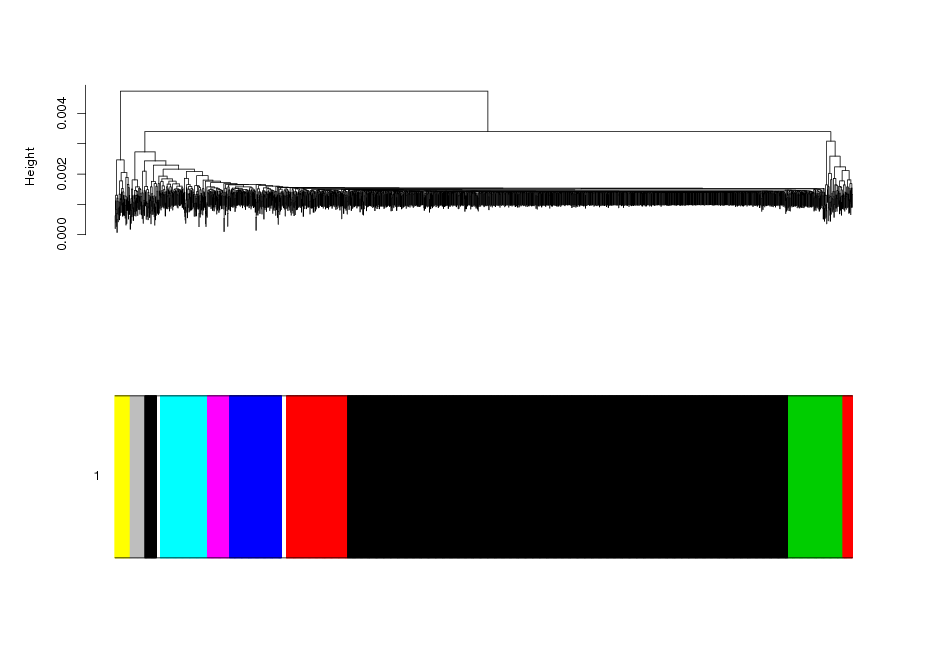


Dataset D6: Huang et al. (2003)


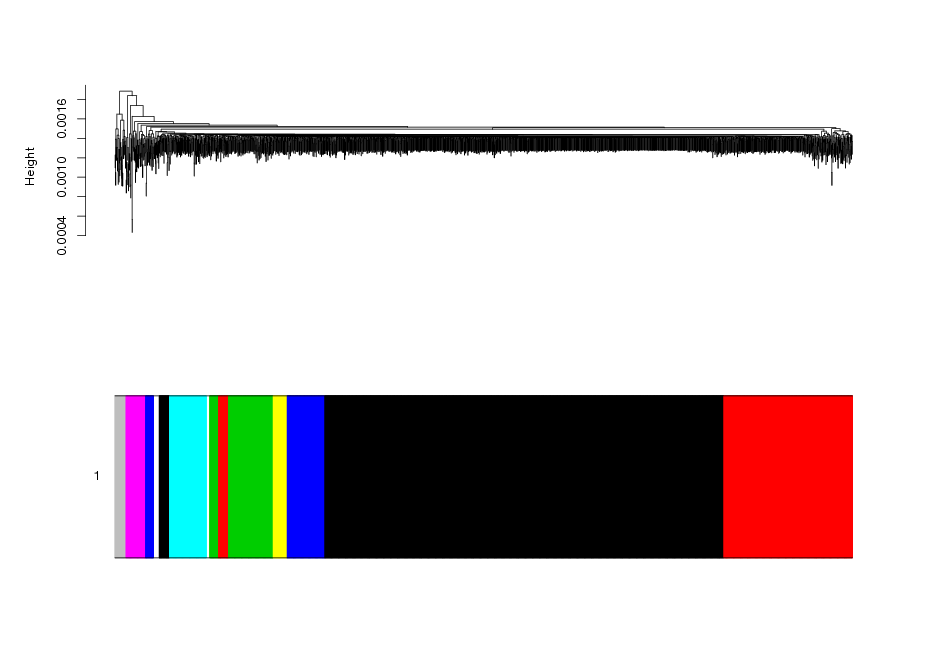

Supplement: Additional file 1 — Results on network module construction. This additional file contains the details on the network modules constructed using WGCNA. [file 1755-8794-4-5-S1.DOC]
